# Position-specific workload and performance analysis in professional rugby union: Insights from global positioning system data and principal component analysis

**DOI:** 10.1371/journal.pone.0332500

**Published:** 2025-10-08

**Authors:** Xiangyu Ren, Simon Boisbluche, Kilian Philippe, Mathieu Demy, Zhiwen Hu, Shuzhe Ding, Jacques Prioux

**Affiliations:** 1 Sino-French Joint Research Center of Sport Science, Key Laboratory of Adolescent Health Assessment and Exercise Intervention of Ministry of Education, College of Physical Education and Health, East China Normal University, Shanghai, China; 2 Movement, Sport, Health Laboratory, Rennes 2 University, Bruz, France; 3 Department of Sports Sciences and Physical Education, École Normale Supérieure de Rennes, Bruz, France; 4 Rugby Club Vannes, French Rugby Federation, Vannes, France; 5 Laboratory of Movement, Balance, Performance and Health, University of Pau and Pays de l’Adour, Tarbes, France; Universidade Federal de Goias, BRAZIL

## Abstract

Quantifying workload and performance is a systematic approach employed by practitioners to enhance their understanding of the training process as a whole. This study aims to utilize data collected from global positioning system (GPS) and video analysis to assess the movement patterns and key performance indicators (KPIs) of players across various positions during both training and match play. Additionally, it seeks to simplify workload analysis by using principal component analysis (PCA) for dimensionality reduction. Over three seasons, data were collected from 63 professional rugby union players, divided into six positional groups: front row, second row, back row, scrum-half, inside backs, and outside backs. The results showed significant positional differences in movement characteristics (p < 0.05, effect size = 0.02–0.59) and KPIs (p < 0.05, effect size = 0.04–0.77). Scrum-halves demonstrated the highest workload in medium and low-intensity activities, while outside backs excelled in high-intensity metrics, and front row forwards consistently had the lowest workload. Regarding KPIs, forwards recorded the most tackles, the highest count of arrivals at offensive and defensive rucks, while scrum-halves accounted for the most kicks, passes, and receipts. In the analysis of training and match workload, PCA extracted two principal components, explaining 73.9% and 79.1% of the variance, respectively. Overall, backs exhibited a higher training workload compared to the forwards, with scrum-halves having the highest total workload during matches. This study demonstrates significant positional differences in key movement variables, providing critical insights into the demands placed on players in different positions.

## Introduction

Rugby union is a sport that incorporates high-intensity intermittent movement, characterized by alternating periods of intense static exertions and collisions with high-speed running, low-intensity movements, and rest intervals [1,2]. Given these physical and tactical demands, coaches and sports scientists employ various techniques to monitor workload and tailor specific training programs for optimal results. Workload refers to the cumulative effect of physiological, psychological, and mechanical stressors experienced by an athlete over a specific period [3]. Workload is categorized as external workload, reflecting physical work performed by the athlete regardless of individual physiological traits, and internal workload, which encompasses psychological and physiological responses to prescribed training exercises [4].

The integration of video-based systems to analyze player movements, combined with GPS technology, enables precise quantification of workload and movement demands during training and competition [5]. Several studies have examined workload in rugby union using these methods, highlighting significant differences in match and training activities across player positions [6,7]. Research shows that workload metrics vary by position during a typical game week, with backs experiencing higher workloads, particularly in terms of distance covered in high-speed and very high-speed running zones [8]. Furthermore, key performance indicators (KPIs) are employed to assess player contributions in specific game aspects, reflecting critical components of match success. KPIs are defined as action variables that objectively capture certain aspects of performance, aiding in workload quantification [9,10]. Previous studies consistently report that forwards endure higher contact workload, engaging in scrums, rucks, tackles, and mauls, whereas backs are more involved in running and open-field play [11,12]. Additionally, differences emerge when positional roles are more finely categorized. For instance, loose forwards experience the highest contact demands, accompanied by greater player workload and distances covered compared to tight forwards [13].

Accurate workload monitoring and optimization are critical for enhancing athlete performance. However, many workload metrics exhibit strong correlations or are challenging to interpret due to interrelated variations. These relationships are influenced by training patterns [14]. Principal component analysis (PCA) addresses this issue by reducing data redundancy. PCA transforms original metrics into uncorrelated principal components (PCs), each representing distinct variance sources (16). This approach simplifies complex datasets, offering clear visualizations of multivariate data in low-dimensional spaces [15,16]. By isolating key variables, PCA provides an efficient, concise representation of workload information, aiding practitioners in understanding and managing athlete workload [17].

While some studies have explored positional differences in pre-season workload [6] or variations in training and match demands throughout a season [8,18], there remains a gap in research comprehensively addressing positional roles, training workloads, and KPIs using PCA [14,19]. This study aimed to investigate (Ⅰ) training and match workload, along with KPIs, across specific positional roles in professional rugby union, including forwards (front row, second row, back row) and backs (scrum-half, inside backs, outside backs), and (Ⅱ) the application of PCA to consolidate workload data, enabling better understanding of positional demands. We hypothesized that (Ⅰ) players in six positional roles would exhibit significant differences in training workload, match workload, and KPIs, and (Ⅱ) PCA would effectively reduce workload complexity, consolidating key metrics into interpretable components.

## Materials and methods

### Experimental approach to the problem

To study position-specific movement demands in professional rugby union players using GPS data and KPIs, we selected data from the 2021–2022, 2022–2023, and 2023–2024 seasons. Given that match days varied, sometimes occurring on Fridays and occasionally on other days of the week, only data from weeks meeting specific criteria were included (22 training weeks, 7 from 2021–2022, 6 from 2022–2023, and 9 from 2023–2024). These weeks featured training sessions on Monday, Tuesday, and Thursday, with matches scheduled on Friday. If players were absent on any of these three training days, their data for that week were excluded from analysis. For KPI analysis, only home games (37 matches, 13 from 2021–2022, 14 from 2022–2023, and 10 from 2023–2024) were included to minimize potential external factors influencing away-game performance, and only players participating for the full 80 minutes were analyzed. In contrast, all players with available GPS data were included in the match workload analysis, regardless of playing time, with workload expressed relative to minutes played (min^-1^) to account for individual differences in match duration, particularly among forwards who were more frequently substituted.

### Participants

Sixty-three male professional rugby union players (mean age: 25.7 ± 5.1 years; height: 190.0 ± 10.0 cm; weight: 103.4 ± 15.8 kg) from a French second-division team (Pro D2) participated in this study. They were grouped into six positional categories based on their primary playing position during the season: front row (props and hooker), second row (locks), back row (flankers and number 8), scrum-half, inside backs (fly-half and centers), and outside backs (wings and full-back) [1]. The forwards group comprised the front row, second row, and back row, while the backs included the scrum-half, inside backs, and outside backs. All participants were informed about the monitoring procedures involved in the study. In accordance with the Declaration of Helsinki, players consented to data collection and use for research purposes, understanding the potential benefits of the study. The training sessions were supervised by professional coaches, who also acted as witnesses to the oral consent process. This study strictly adhered to ethical standards set by the University of Rennes and affiliated research laboratories.

### Procedures

#### Workload monitoring.

Workload data were collected using the Vector X7 GPS device (Catapult Innovations, Australia), which includes integrated 10 Hz GPS, a 100 Hz triaxial accelerometer, a gyroscope, and a 100 Hz magnetometer. To ensure proper satellite connectivity, devices were activated 30 minutes before field training in an open area. Each player wore a specially designed vest housing the sensor unit (81 × 43 × 16 mm, 53 g) positioned between the shoulder blades. This device has been validated in previous research for tracking running and acceleration metrics in team sports [20,21].

Data from GPS and inertial sensors were exported using OpenField Console 3.7 software and stored in the OpenField Cloud for further analysis. Metrics included total distance (TD), player load (PL), acceleration and deceleration counts within specific speed thresholds (e.g., 2–2.5 m·s^-2^, 2.5–3 m·s^-2^, > 3 m·s^-2^), medium-speed running (MSR: 15–18 km·h^-1^), high-speed running (HSR: 18–21 km·h^-1^), very high-speed running (VHSR: 21–25 km·h^-1^), sprint running (SR: > 25 km·h^-1^), and repeated high-intensity efforts (RHIE) (Table 1).

**Table 1:** Global positioning system variables and key performance indicators [5,22,23].

	Variables	Units	Definition
Global positioning system (GPS) metrics	Total distance (TD)	m	Assessed from GPS, correspond to the total distance covered by the players during the ball-in-play time of training.
	Acceleration zone 1 (AZ1)	count	The number of accelerations between 2 and 2.5 m·s^-2^.
	Acceleration zone 2 (AZ2)	count	The number of accelerations between 2.5 and 3 m·s^-2^.
	Acceleration zone 3 (AZ3)	count	The number of accelerations above 3 m·s^-2^.
	Deceleration zone 1 (DZ1)	count	The number of decelerations between 2 and 2.5 m·s^-2^.
	Deceleration zone 2 (DZ2)	count	The number of decelerations between 2.5 and 3 m·s^-2^.
	Deceleration zone 3 (DZ3)	count	The number of decelerations above 3 m·s^-2^.
	Player load (PL)	Arbitrary unit	A modified vector magnitude expressed as the square root of the sum of the squared instantaneous rates of change in acceleration in each of the three orthogonal planes and divided by 10.
	Medium-speed running (MSR)	m	Distance covered between 15 and 18 km·h^-1^.
	High-speed running (HSR)	m	Distance covered between 18 and 21 km·h^-1^.
	Very high-speed running (VHSR)	m	Distance covered between 21 and 25 km·h^-1^.
	Sprint running (SR)	m	Distance covered above 25 km·h^-1^.
	Repeated high-intensity efforts (RHIE)	count	Three consecutive high-intensity efforts (contact, acceleration, or sprint) occurring within 21 s.
Key performance indicator (KPI)	Total complete tackle	count	An event where a player carrying the ball (the ball-carrier) is physically impeded by another player (the tackler).
	Carries	count	Counts of times the player being in possession of the ball when being tackled by a defending player and included instances whereby the ball carrier offloaded the ball in the process of being tackled.
	Meters carried	m	Total meters carried past the gain line.
	Total kicks	count	Counts of times the player kicks the ball.
	Kick meters	m	The distance a player kicks during a match.
	Total turnovers	count	Counts of times a player turns over the ball into an offensive situation from a defensive play.
	Total passes	count	Counts of times the player successful and unsuccessful passes the ball.
	Total receipts	count	Counts of times the attacking player successfully catches or picks up the ball.
	Total OOA	count	Count of times defensive players arrive at the offensive and the defensive ruck.
	Total OOAA	count	Count of times offensive players arrive at the offensive ruck.
	Total OOAD	count	Count of times defensive players arrive at the defensive ruck.

#### Game key performance indicators.

On-field activity during games was recorded in a performance matrix using video footage analyzed by Opta (Stats Perform, Pro Rugby Hub). Analysts collected data in real-time, conducting thorough accuracy checks. Each analyst underwent 3–6 months of training before coding games independently. KPIs analyzed in this study (Table 1) included the total number of complete tackles, carries, kicks, turnovers, passes, receipts. Additional metrics included the count of times defensive players arrived at both offensive and defensive rucks (overall arrivals, OOA), offensive ruck arrivals (OOAA), and defensive ruck arrivals (OOAD), as well as meters carried, and meters kicked.

### Statistical analysis

#### Analysis of variance.

Results were expressed as means ± standard deviation (SD). Analysis of variance (ANOVA) was conducted using SPSS (version 27; SPSS Inc., Chicago, IL), with significance set at p < 0.05. The Shapiro-Wilk test assessed data normality, and homogeneity of variance was checked. Where required, non-parametric Kruskal-Wallis one-way ANOVA tests were performed. Effect size (ES) was measured using ε², with thresholds set as 0.01 (small), 0.08 (medium), and 0.26 (large) [24]. One-way ANOVAs were also performed to assess seasonal differences. As only small effects were observed, season was not included as a covariate in the final analysis.

#### Principal component analysis.

Metrics used for training workload PCA were weekly TD, AZ1, AZ2, AZ3, DZ1, DZ2, DZ3, PL, MSR, HSR, VHSR, SR, RHIE, and metrics used for match workload included m·min^-1^, PL·min^-1^, RHIE·min^-1^, counts of accelerations and decelerations above 2 and 2.5 m·s^-2^·min^-1^, distance for velocities greater than (>) 15, 18, 21, 25 km·h^-1^ (>MSR·min^-1^, > HSR·min^-1^, > VHSR·min^-1^, SR·min^-1^;). The suitability of the data for PCA was evaluated using the Kaiser-Meyer-Olkin (KMO) measure of sampling adequacy and Bartlett’s test of sphericity. A KMO value of 0.6 or higher was considered sufficient to proceed with PCA. In this study, the KMO for training workload was 0.91 and for game workload 0.83, indicating strong suitability for PCA. Eigenvalues greater than 1 were considered for the extraction of principal component (PC).

In PCA, the original variables were first standardized so that they had a mean of 0 and a standard deviation of 1. Then, based on the standardized data matrix, the covariance matrix was computed and subjected to eigenvalue decomposition, resulting in eigenvalues and eigenvectors. The number of significant principal components was determined by a conventional criterion, selecting only those that explained at least 70% of the cumulative variance [25]. For each retained PC, only the original workload variables with a loading greater than 0.7 were kept [26]. The PC loading matrix can be obtained by dividing the factor loading matrix by the square root of the corresponding eigenvalues, as follows [16]:


$$\text{Principal component loading matrix}=\frac{\text{Factor loading matrix}}{\sqrt{\lambda_{\text{i}}}}$$

where $\lambda_{i}$ is the eigenvalue corresponding to the i-th PC. The eigenvectors form the PC loading matrix, with each eigenvector representing the direction of a PC and reflecting the projection of the original variables onto the PCs. Next, the PC scores were calculated by multiplying the standardized variable matrix $X_{\text{std}}$ by the PC loading matrix, yielding the scores for each sample on the PCs:


$$Y=X_{\text{std}}\cdot L$$

where L is the PC loading matrix, and Y is the matrix of PC scores.

For the first two PCs, PC1 and PC2, the scores were calculated as follows:


$$Y_{\text{1}}=X_{std}\cdot L_{\text{1}},\;Y_{\text{2}}=X_{std}\cdot L_{\text{2}}$$

Finally, the comprehensive score can be obtained through a weighted sum, where the weights were determined by the variance explained by each PC, and normalized as follows:


$$Y_{\text{total}}=W_{\text{1}}\cdot Y_{\text{1}}+W_{\text{2}}\cdot Y_{\text{2}}$$

The weights $W_{\text{1}}$ and $W_{\text{2}}$ represent the variance contribution ratios of PC1 and PC2, respectively. After normalization, the weights are computed using the following formula:


$$W_{\text{1}}=\frac{{\text{Variance contribution ratio}}_{PC\text{1}}}{\text{Cumulative variance contribution ratio}},\;W_{\text{2}}=\frac{{\text{Variance contribution ratio}}_{PC\text{2}}}{\text{Cumulative variance contribution ratio}}$$

PCA was performed using both R v4.2.3 (R Foundation for Statistical Computing, Vienna, Austria) and SPSS. Figures were generated using R and GraphPad Prism (GraphPad Software 9).

## Results

### Weekly training workload

Table 2 presents the weekly training workload of players across six positional groups. Backs consistently covered more total distance (TD) than forwards, with the front row displaying the lowest values among all groups (p < 0.05, ES = 0.18). Acceleration and deceleration metrics also varied significantly between positions. AZ1 values for the front row and second row were lower than those of the backs, with the front row showing the lowest levels (p < 0.05, ES = 0.09). Inside backs demonstrated the highest values for AZ2, while outside backs excelled in AZ3, significantly surpassing the forwards and scrum-halves. The scrum-half showed higher DZ1 metrics compared to forwards (p < 0.05, ES = 0.10–0.32), while DZ2 values were higher for backs than forwards (p < 0.05, ES = 0.25). Outside backs exhibited the highest DZ3 metrics among all positions (p < 0.05, ES = 0.40). Scrum-halves recorded the highest values for PL and MSR (p < 0.05, ES = 0.02 and 0.28). Backs consistently outperformed forwards in high-speed running zones (HSR, VHSR, SR) and repeated high-intensity efforts (RHIE), with outside backs exceeding inside backs in all metrics (p < 0.05, ES = 0.45–0.55). Among forwards, back rows demonstrated significantly greater workloads than front rows in all metrics except PL. Second rows likewise exceeded front rows in most metrics, with no significant differences observed in AZ2 and PL.

**Table 2:** Comparison of weekly training workload of players at six positions.

	Position						ES
	Forward			Back			
	Front row	Second row	Back row	Scrum-half	Inside backs	Outside backs	ε²
TD (m)	7981.0 ± 1588.7	8862.2 ± 1654.6 ^a^	9054.8 ± 1714.8 ^b^	9768.6 ± 1669.9 ^c,g,j^	9938.2 ± 2171.2 ^d,h,k^	10235.7 ± 2101.8 ^e,i,l^	0.18
AZ1 (n)	56.3 ± 14.3	61.4 ± 16.0 ^a^	64.1 ± 14. 4 ^b^	70.9 ± 16.0 ^c,g^	67.8 ± 16.8 ^d,h^	63. 5 ± 15.7 ^e,i^	0.09
AZ2 (n)	35.3 ± 11.4	39.3 ± 11.34	41.2 ± 10.7 ^b^	45.0 ± 12.2 ^c,g^	46.5 ± 11.7 ^d,h,k^	44.6 ± 10.9 ^e,i^	0.12
AZ3 (n)	21.3 ± 11.1	28.6 ± 11.6 ^a^	30.9 ± 12.2 ^b^	34.8 ± 13.5 ^c^	42.8 ± 15.4 ^d,h,k,m^	44.8 ± 15.0 ^e,i,l,n^	0.32
DZ1 (n)	43.6 ± 13.1	50.3 ± 14.6 ^a^	51.6 ± 11.2 ^b^	59.3 ± 13.5 ^c,g,j^	54.8 ± 16.0 ^d^	54.1 ± 15.6 ^e^	0.10
DZ2 (n)	21.6 ± 9.2	27.0 ± 9.4 ^a^	29.2 ± 7.8 ^b^	35.5 ± 7.3 ^c,g,j^	34.0 ± 10.6 ^d,h,k^	34.7 ± 11.0 ^e,i,l^	0.25
DZ3 (n)	14.6 ± 9.4	22.6 ± 12.2 ^a^	25.5 ± 9.5 ^b,f^	32.3 ± 12.4 ^c,g,j^	35.7 ± 14.9 ^d,h,k^	41. 9 ± 15.8 ^e,i,l,n,o^	0.40
PL (AU)	891.4 ± 165.8	878.1 ± 166.7	919.9 ± 199.2	958.6 ± 182.4	927.7 ± 188.6	951.9 ± 175.8 ^e,i^	0.02
MSR (m)	634.1 ± 224.2	721.9 ± 212.0 ^a^	824.0 ± 228.6 ^b,f^	1113.0 ± 232.6 ^c,g,j,m,n^	931.7 ± 252.9 ^d,h,k^	916.6 ± 240.9 ^e,i,l^	0.28
HSR (m)	288.7 ± 138.3	386.3 ± 194.4 ^a^	486.5 ± 144.2 ^b,f^	672.8 ± 171.7 ^c,g,j^	600.8 ± 217.9 ^d,h,k^	674.4 ± 210.7 ^e,i,l,o^	0.45
VHSR (m)	115.6 ± 82.2	186.6 ± 119.2 ^a^	259.4 ± 132.1 ^b,f^	359.0 ± 150.5 ^c,g,j^	392.8 ± 177.9 ^d,h,k^	554.1 ± 191.8 ^e,i,l,n,o^	0.55
SR (m)	17.5 ± 29.8	38.5 ± 40.7 ^a^	51.7 ± 59.4 ^b^	70.2 ± 52.2 ^c,g,j^	124.4 ± 114.0 ^d,h,k,m^	233.4 ± 155.0 ^e,i,l,n,o^	0.47
RHIE (n)	17.4 ± 9.6	25.1 ± 11.3 ^a^	30.0 ± 9.9 ^b,f^	35.8 ± 9.8 ^c,g,j^	36.2 ± 12.5 ^d,h,k^	43.6 ± 12.55 ^e,i,l,n,o^	0.41

^a^Front row ***vs.*** second row; ^b^ front row ***vs.*** back row; ^c^ front row ***vs.*** scrum-half; ^d^ front row ***vs.*** inside backs; ^e^ front row ***vs.*** outside backs; ^f^ second row ***vs.*** back row; ^g^ second row ***vs.*** scrum-half; ^i^ second row ***vs.*** outside backs; ^j^ back row ***vs.*** scrum-half; ^k^ back row ***vs.*** inside backs; ^l^ back row ***vs.*** outside backs; ^m^ scrum-half ***vs.*** inside backs; ^n^ scrum-half ***vs.*** outside backs. p < 0.05.

### Match workload

The relative match workload of players is summarized in Table 3. Scrum-halves exhibited significantly higher values for m·min^-1^, PL·min^-1^, and>MSR·min^-1^ compared to other positions (p < 0.05, ES = 0.24–0.55). Forwards, particularly front and second row players, recorded lower match workloads than back rows and backs (p < 0.05, ES = 0.42–0.59). Among backs, outside backs had greater workload in>HSR·min^-1^ and>VHSR·min^-1^ compared to inside backs (p < 0.05, ES = 0.59 and 0.58).

**Table 3:** Comparison of relative workload of players at six positions during match.

	Position						ES
	Forward			Back			
	Front row	Second row	Back row	Scrum-half	Inside backs	Outside backs	ε²
m·min^-1^	102.0 ± 12.8	110.0 ± 10.0 ^a^	111.0 ± 8.9 ^b^	124.0 ± 13.6 ^c,g,j,m,n^	114.0 ± 12.8 ^d,h,k,o^	110.0 ± 9.0 ^e^	0.24
PL·min^-1^	15.1 ± 2.1 ^d,e^	15.3 ± 6.5 ^h,i^	14.7 ± 2.1 ^k,l^	16.1 ± 1.7 ^c,g,j,m,n^	12.6 ± 1.9	12.4 ± 1.8	0.33
RHIE·min^-1^	0.3 ± 0.2	0.4 ± 0.2 ^a^	0.7 ± 0.2 ^b,f^	0.7 ± 0.2 ^c,g^	0.7 ± 0.2 ^d,h^	0.7 ± 0.2 ^e,i^	0.42
Acc + Dec > 2·min^-1^	2.4 ± 0.8	2.9 ± 0.8 ^a^	3.6 ± 0.6 ^b,f^	4.5 ± 1.1 ^c,g,j^	4.0 ± 0.8 ^d,h,k^	3.6 ± 0.6 ^e,i,l^	0.46
Acc + Dec > 2.5·min^-1^	1.1 ± 0.5	1.4 ± 0.5 ^a^	2.1 ± 0.5 ^b,f^	2.4 ± 0.8 ^c,g^	2.5 ± 0.7 ^d,h,k^	2.3 ± 0.5 ^e,i,l^	0.53
>MSR·min^-1^	14.8 ± 7.2	19.3 ± 7.0 ^a^	28.0 ± 7.2 ^b,f^	39.9 ± 12.2 ^c,g,j,m,n^	31.3 ± 8.0 ^d,h,k^	32.8 ± 6.8 ^e,i,l^	0.55
>HSR·min^-1^	6.3 ± 4.2	8.1 ± 4.4 ^a^	15.1 ± 5.6 ^b,f^	22.3 ± 9.5 ^c,g,j^	18.8 ± 6.4 ^d,h,k^	22.1 ± 5.2 ^e,i,l,o^	0.59
>VHSR·min^-1^	2.3 ± 2.4	3.1 ± 2.3 ^a^	6.5 ± 3.6 ^b,f^	9.7 ± 5.7 ^c,g,j^	10.0 ± 4.7 ^d,h,k^	13.6 ± 4.0 ^e,i,l,n,o^	0.58
SR·min^-1^	0.2 ± 0.6	0.3 ± 0.7 ^a^	1.1 ± 1.4 ^b,f^	2.1 ± 2.6 ^c,g^	2.9 ± 2.3 ^d,h,k,m^	5.4 ± 2.6 ^e,i,l,n^	0.59

^a^Front row ***vs.*** second row; ^b^ front row ***vs.*** back row; ^c^ front row ***vs.*** scrum-half; ^d^ front row ***vs.*** inside backs; ^e^ front row ***vs.*** outside backs; ^f^ second row ***vs.*** back row; ^g^ second row ***vs.*** scrum-half; ^h^ second row ***vs.*** inside backs; ^i^ second row ***vs.*** outside backs; ^j^ back row ***vs.*** scrum-half; ^k^ back row ***vs.*** inside backs; ^l^ back row ***vs.*** outside backs; ^m^ scrum-half ***vs***. inside backs; ^n^ scrum-half ***vs.*** outside backs; ^o^ inside backs ***vs***. outside backs. p < 0.05.

### Key performance indicators

Table 4 compares the KPIs of players across positions. Second row and back row players recorded significantly higher tackle counts than backs (p < 0.05, ES = 0.45). Among forwards, back rows achieved the highest carry counts (p < 0.05, ES = 0.04). Outside backs recorded the most meters carried, outperforming other groups, while inside backs surpassed both forwards and the scrum-half in this metric (p < 0.05, ES = 0.18). Scrum-halves had the highest totals for kicks, kick meters, passes, and receipts (p < 0.05, ES = 0.123–0.77). Front row players had the fewest turnovers (p < 0.05, ES = 0.04). Forwards dominated OOA, OOAA, and OOAD metrics compared to backs, with the second row achieving the highest OOA and OOAA counts (p < 0.05, ES = 0.20–0.61).

**Table 4:** Comparison of key performance indicators of players at six positions.

	Position						ES
	Forward			Back			
	Front row	Second row	Back row	Scrum-half	Inside backs	Outside backs	η²
Total Complete tackle	7.3 ± 1.5	10.1 ± 4.3 ^g,h,i^	11.4 ± 5.2 ^j,k,l^	7.2 ± 3.4 ^n^	6.1 ± 3.0 ^o^	3.5 ± 2.1	0.45
Carries	4.3 ± 2.1	5.8 ± 3.7	8.1 ± 4.4 ^f,k^	6.9 ± 2.8	6.6 ± 3.0	7.6 ± 3.5 ^i,o^	0.04
Meters Carried	17.3 ± 10.1	25.8 ± 25.2	40.7 ± 26.8 ^f^	27.4 ± 22.9	49.0 ± 26.3 ^h,k,m^	68.3 ± 38.4 ^i,l,n,o^	0.18
Total Kicks	–	–	1.2 ± 0.4	10.8 ± 5.2 ^j,m,n^	4.6 ± 4.6 ^k,o^	3.1 ± 2.4 ^l^	0.21
Kick Meters	–	–	35.3 ± 26.3	294.1 ± 150.8 ^j,m,n^	151. 2 ± 165.6 ^k,o^	106.0 ± 91.5 ^l^	0.12
Total Turnovers	1.0 ± 0.0	1.4 ± 0.5 ^a^	1.4 ± 0.6 ^b^	1.5 ± 0.5 ^c^	1.7 ± 0.9 ^d,k^	1.8 ± 1.0 ^e,i,l^	0.04
Total Passes	2.7 ± 2.9	3.8 ± 2.2	5.0 ± 3.0 ^b,f,l^	63.3 ± 13.6 ^g,j,m,n^	7.7 ± 7.0 ^h,k,o^	3.7 ± 2. 8	0.77
Total Receipts	7.3 ± 4.2	8.1 ± 4.2	11.3 ± 5.4 ^f^	77.4 ± 13.6 ^c,g,j,m,n^	15.5 ± 10.1 ^h,k,o^	12.3 ± 5.9 ^i^	0.63
Total OOA	19.0 ± 3.5	22.9 ± 6.7 ^f,g,h,i^	18.0 ± 6.1 ^j,k,l^	4.8 ± 2.8	8.6 ± 4.6 ^m,o^	6.5 ± 3.3	0.61
Total OOAA	14.7 ± 2.9	19.6 ± 6.7 ^f,g,h,i^	13.4 ± 5.3 ^j,k,l^	3.2 ± 2.5	6.7 ± 3.7 ^m,o^	5.0 ± 2.7	0.58
Total OOAD	4.3 ± 0.6	3.6 ± 2.3 ^h,i^	4.9 ± 3.1 ^f,j,k,l^	3.2 ± 1.7	2.6 ± 2.0 ^o^	2.1 ± 1.5	0.20

^a^Front row ***vs.*** second row; ^b^ front row ***vs.*** back row; ^c^ front row ***vs.*** scrum-half; ^d^ front row ***vs.*** inside backs; ^e^ front row ***vs.*** outside backs; ^f^ second row ***vs.*** back row; ^g^ second row ***vs.*** scrum-half; ^h^ second row ***vs.*** inside backs; ^i^ second row ***vs.*** outside backs; ^j^ back row ***vs.*** scrum-half; ^k^ back row ***vs.*** inside backs; ^l^ back row ***vs.*** outside backs; ^m^ scrum-half ***vs***. inside backs; ^n^ scrum-half ***vs.*** outside backs; ^o^ inside backs ***vs***. outside backs. p < 0.05.

### Dimensionality reduction for training workload

**Fig 1:**
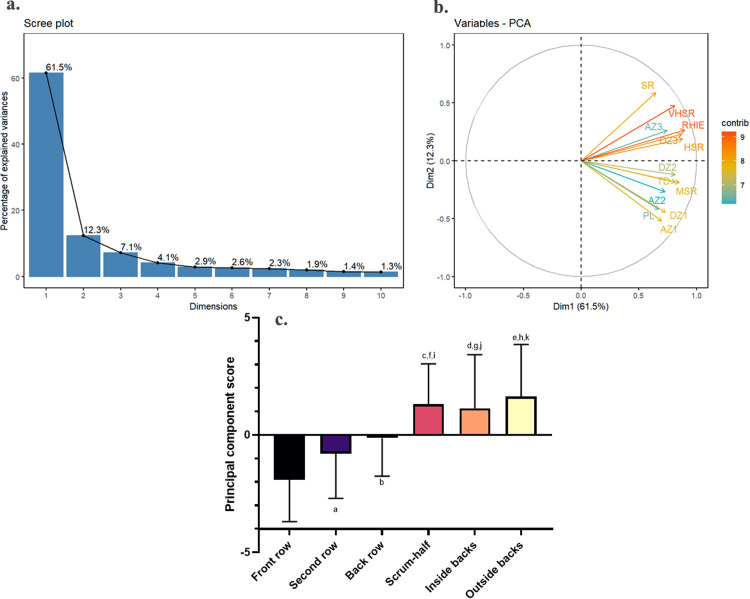
Principal component analysis of weekly training workload in rugby union players. a: Scree plot of eigenvalues from principal component analysis of weekly training workload. b: Graphical output of principal component analysis using the first two dimensions (dim1 and dim2) of weekly training workload. A correlation circle displaying the original variables as vectors in a 2-dimensional space created by the first two principal components (PCs). The length and color of each vector represent the variable’s overall contribution, while the projection of the vector onto a principal component shows its contribution to that specific component. The contributions are based on the squared cosine of the angle. c: Comparison of principal component analysis scores by positions based on weekly training workload. ^a^ Front row ***vs.*** second row; ^b^ front row ***vs.*** back row; ^c^ front row ***vs.*** scrum-half; ^d^ front row ***vs.*** inside backs; ^e^ front row ***vs.*** outside backs; ^f^ second row ***vs.*** scrum-half; ^g^ second row ***vs.*** inside backs; ^h^ second row ***vs.*** outside backs; ^i^ back row ***vs.*** scrum-half; ^j^ back row ***vs.*** inside backs; ^k^ back row ***vs.*** outside backs. p < 0.05, ε² = 0.26.

PCA identified two principal components (PCs) for training workload. PC1, with an eigenvalue of 7.996, explained 61.5% of the variance, while PC2, with an eigenvalue of 1.605, explained 12.3% of the variance. Combined, the PCs accounted for 73.8% of total variance (Fig 1a). PC1 was dominated by high-intensity metrics, including AZ3, DZ3, HSR, VHSR, SR, and RHIE, representing “high-intensity explosive workload”. PC2, characterized by AZ1, AZ2, DZ1, DZ2, MSR, TD, and PL, represented “low-intensity continuous workload” (Fig 1b). Weekly PC scores revealed that front row players consistently had the lowest training workload among all groups, while backs had higher scores than forwards overall (p < 0.05, ES = 0.32; Fig 1c).

### Dimensionality reduction of relative workload for matches

**Fig 2:**
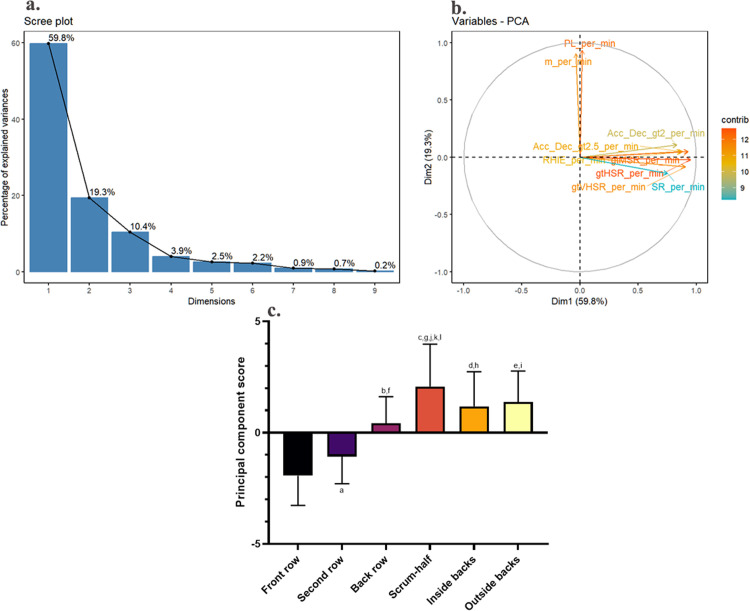
Principal component analysis of relative match workload in rugby union players. a: Scree plot of eigenvalues from principal component analysis of relative match workload. b: Graphical output of principal component analysis using the first two dimensions (dim1 and dim2) of relative match workload. A correlation circle displaying the original variables as vectors in a 2-dimensional space created by the first two principal components (PCs). The length and color of each vector represent the variable’s overall contribution, while the projection of the vector onto a principal component shows its contribution to that specific component. The contributions are based on the squared cosine of the angle. c: Comparison of principal component analysis scores by positions based on relative match workload. ^a^ Front row ***vs.*** second row; ^b^ front row ***vs.*** back row; ^c^ front row ***vs.*** scrum-half; ^d^ front row ***vs.*** inside backs; ^e^ front row ***vs.*** outside backs; ^f^ second row ***vs.*** scrum-half; ^g^ second row ***vs.*** inside backs; ^h^ second row ***vs.*** outside backs; ^i^ back row ***vs.*** scrum-half; ^j^ back row ***vs.*** inside backs; ^k^ back row ***vs.*** outside backs. p < 0.05, ε² = 0.54.

Figs 2a and 2b illustrate the dimensionality reduction results for relative match workload. PC1 accounted for 59.8% of variance and was interpreted as “overall match performance”, as most GPS metrics loaded heavily onto this component, except m·min^-1^ and PL·min^-1^. PC2 explained 19.3% of variance and was dominated by m·min^-1^ and PL·min^-1^, which together represent “locomotor workload” [27]. Scrum-halves exhibited the highest overall PC scores, indicating the greatest match workload, while front row and second row players had the lowest scores (p < 0.05, ES = 0.54; Fig 2c).

## Discussion

The primary aim of this study was to examine the training and match workload, as well as the performance (i.e., KPIs), of professional rugby union players across multiple seasons. Additionally, the study explored positional differences and employed PCA to simplify workload metrics. This process involved: (Ⅰ) comparing cumulative training workload during match weeks across positions, (Ⅱ) comparing match workload between positions, (Ⅲ) evaluating KPIs across positions, and (Ⅳ) using PCA to reduce workload dimensionality and calculate overall scores. The results indicated that, except for PL, front row players consistently had the lowest training workload, while scrum-halves exhibited higher workload in low-to-moderate intensity activities, and outside backs had the highest workload during high-intensity efforts. Overall, backs had higher cumulative training workload scores than forwards. During matches, scrum-halves covered the most distance and had the highest PL and MSR, while outside backs again showed the highest workload in high-intensity activities. In contrast, apart from m·min^-1^ and PL·min^-1^, front row and second row players recorded the lowest match workload. Scrum-halves had the highest overall match workload scores. In terms of KPIs, forwards recorded more tackles and total OOA, scrum-halves made more kicks, kick meters, passes, and receipts, while outside backs carried the ball for greater distances. The study also introduced new composite variables to define performance, identifying two key indicators from GPS data during training: “high-intensity explosive workload” and “low-intensity continuous workload”. Similarly, two new performance metrics were derived from match GPS data: “overall performance” and “locomotor workload”.

Workload monitoring is a key focus in an elite team sport environment. Bradley et al. [28] investigated in-season weekly training workload for forwards and backs in an elite European rugby union squad and found that backs ran significantly more total weekly distance than forwards, covering more standing, walking, jogging, high-speed running, and sprinting. Additionally, backs completed more accelerations than forwards (p < 0.01). These findings align with our results, showing that these differences can be attributed to the distinct training tasks between forwards and backs in rugby union. For example, forwards were more involved in tackling, scrumming, mauls, and line-outs, whereas backs focused more on accelerating and running with the ball. However, Bradley et al. [28] also found that there was no significant difference in the number of RHIE per week between forwards (19.2 ± 7.9) and backs (15.4 ± 10.3) in training. Our study expanded on this by comparing specific positional groups within forwards and backs. Specifically, outside backs had a significantly higher number of RHIE (43.57 ± 12.50) than forwards and other backs, while front row players recorded the fewest RHIE. This positional breakdown suggests that large differences may exist not only between forwards and backs but also among specific positions, likely due to variations in playing style, tactical arrangements, and training methods across teams or leagues.

Studies on workload monitoring during matches are prevalent in professional rugby union. They have shown that backs have higher match workloads than forwards, both in terms of total distance run, relative distance, and activity in various speed zones between low-speed, high-speed, and very high-speed running [18,29]. Our study supports these results, but it identified workloads higher than those reported in prior findings. Across arbitrary velocity bands, Tee et al. [30] provided a more detailed division of the different positions and found that front and second row players walked and jogged more relative distances in the match, whereas scrum-halves excelled in medium-speed running distances. Inside backs had the highest frequency of sprinting and acceleration (>2.75 m·s⁻^2^). According to the study by Jones et al. [31], inside backs and outside backs experience the highest demands for high-speed running during matches, while loose forwards face the greatest demands for RHIE. However, our results indicated that workload varied significantly from position to position. Front and second row players recorded the lowest workload, while scrum-halves exhibited the highest acceleration and deceleration frequencies (>2 m·s⁻^2^), HSR, and RHIE. These differences may reflect the varying tasks and physical demands that players in different positions perform during the match.

Due to the different physical demands of players in various positions during matches [2,7], KPIs vary significantly across tactical roles. Ungureanu et al. [32] quantified the performance of elite national U20 rugby players over five matches, showing that the median total tackles for forwards was 7, compared to 6 for backs. Similarly, the median number of total passes for forwards was 0.83, while for backs, it was 2. Forwards made no kicks, whereas the median number of kicks for backs was 1. Unlike studies that primarily examine differences between forwards and backs, our study offers a detailed analysis of the match performance of players in six different positions in professional rugby union.

Our analysis found that most tackles were made by second row and back row players, while most passes and kicks were completed by scrum-halves. The number of kicks by scrum-halves was notably higher than those recorded by U20 rugby players, which may be attributed to the differences in competition level. For carries, U20 players showed relatively equal contributions between forwards and backs (median of 3). However, our research found that within the forwards, front row and second row players had fewer carries (4.33 ± 2.08 and 5.77 ± 3.70, respectively), while back row players had significantly more (8.05 ± 4.42). In contrast, the three backline positions demonstrated more balanced carry counts (ranging from 6.55 ± 3.01 to 7.64 ± 3.50). In terms of tackles, another study provided contradictory results, suggesting that backs made more tackles than forwards (10 vs. 12) [33]. According to Smart et al. [34], the number of turnovers between forwards and backs was similar (0.5 ± 1.1), while our study found that inside backs and outside backs had significantly more turnovers than forwards.

Coaches and sports practitioners should plan and implement targeted training programs based on the specific demands of each player’s position. Forwards, especially second row and back row players, are more involved in high-contact actions during possession or near the ball. Training for these players should emphasize high-intensity scenarios to enhance speed and acceleration. Backs, on the other hand, are more involved in off-ball movements such as lateral or backward shifts to readjust defensive positioning. Training for backs should emphasize organization and the ability to reform defensive structures quickly, ensuring effective player distribution and the rapid formation of defensive lines. The focus should remain on overall defensive effectiveness, particularly how to quickly reorganize the defensive structure and reduce unnecessary high-speed running. In tactical training, coaches should encourage forwards to actively cover more ground and motivate backs to maintain the defensive structure, thereby improving the team’s defensive efficiency and physical workload management [32].

Using PCA for training workload management is an emerging method that helps optimize the presentation and visualization of data. This approach allows practitioners to identify and focus on key variables, eliminating redundancy and enhancing efficiency. It enables coaches and decision-makers to monitor the most concise and effective set of variables, making it easier to interpret changes in training workload and their implications for performance. This, in turn, facilitates evidence-based and more precise adjustments [35,36]. Our findings further show how PCA-derived metrics can inform position-specific training strategies. Backs generally showed higher scores on the high-intensity workload component, reflecting greater demands for repeated sprinting and explosive efforts. Their training should therefore emphasize speed, change of direction, and repeated high-intensity actions. In contrast, forwards, particularly front row players, had lower scores on both training and match workload components. This reflects their engagement in collision-based efforts such as scrums and rucks, where high-intensity output occurs mainly in closed-skill contact situations rather than in open-field running. For these players, training should target strength, anaerobic capacity, and controlled aerobic volume. Williams et al. [15] demonstrated the potential of PCA by using session rating of perceived exertion (sRPE) and its derived metrics to optimize the combination of training workload measurements in professional rugby union,. Their findings highlighted the role of PCA in reducing complexity while retaining meaningful information. Weaving et al. [14] combined internal and external metrics for dimensionality reduction, suggesting that if multiple variables are contained within the same PC, selecting just one can effectively quantify training workload. In Rugby League, dimensionality reduction was similarly applied in another study to analyze players’ physical fitness and performance, allowing comparisons between players at different competition levels [19].

In our study, we performed dimensionality reduction on multiple training and match workload and compared the scores of six different positions. To our knowledge, this is the first study to comprehensively compare workload scores across different positions in rugby union. The advantage of this method is that coaches and practitioners no longer need to rely on a single metric when comparing players’ workload. For instance, one player’s value might be higher in one metric but lower in another, making comparisons complex. By calculating a composite PC score, the overall workload for each player becomes much clearer, allowing for more straightforward decision-making.

Despite providing valuable insights into workload comparisons and the application of PCA, this study has several limitations. First, the absence of internal workload data (e.g., heart rate, sRPE) and contact-related metrics (e.g., contact, impact) limits the ability to fully capture workload demands and their associated fatigue impact, particularly for forwards. Forwards are less likely to match backs in terms of distance, acceleration, deceleration, and high-speed running due to their specific physical and tactical roles. This limitation arose from the data collection process, and future studies should include these variables. In addition, the reliability of match event data derived from Opta remains uncertain, as inter- and intra-analyst consistency for rugby-specific coding has not been clearly established [37]. Moreover, the study was conducted using data from a single team, meaning the results may be influenced by specific team characteristics such as player profiles, training styles, and match tactics, which could limit generalizability. Another potential limitation relates to the inclusion criteria, which may have introduced selection bias. KPI analysis was limited to players who completed full 80-minute matches to ensure consistent exposure for assessing performance trends such as fatigue and decision-making under pressure. However, this approach may exclude players with higher rotation frequency, lower fitness, or increased injury susceptibility. While relative workload per minute helps standardize data across players with different match durations, it may overlook key fluctuations in intensity. Averaging across the full match can mask peak efforts and dynamic changes, particularly during high-tempo phases. To address this, future studies could address this by analyzing shorter time intervals to better reflect within-match workload variability. Further research on a more heterogeneous sample that includes multiple teams across different leagues is necessary to validate these findings.

### Practical applications

The findings from this study highlight distinct differences in training and match workload across player positions, providing actionable insights for designing position-specific training and conditioning programs. Backs, particularly outside backs, consistently exhibited higher high-intensity workload, while forwards, especially the front row, demonstrated lower overall demands. Strength and conditioning coaches can use these insights to optimize training regimens by addressing the specific demands of each position to enhance performance. In addition, the long-term average workload metrics derived from multiple seasons offer practical reference points for benchmarking individual players’ training and match exposure. These values can help coaches assess whether players, particularly developing or newly transferred ones, are adapting appropriately to the physical demands of their position. Long-term averages also provide a stable framework for planning when short-term data are unavailable or inconsistent. Through data reduction and variable selection, practitioners in team sports can improve their monitoring and analysis practices. By using PCA to simplify and reduce dimensionality, practitioners can monitor and interpret training and match workload more efficiently, focusing on key high-intensity and low-intensity workload metrics. This approach provides a structured framework for managing workload across different positions, facilitating tailored training plans that reflect the specific demands of forwards and backs. By replacing the need to track numerous raw GPS variables on a daily basis, coaches and sports science staff can instead focus on core composite scores: “high-intensity explosive workload” and “low-intensity continuous workload” for training, as well as “overall match performance” and “locomotor workload” for matches. This greatly simplifies data interpretation and streamlines the decision-making process. Over time, PC scores can serve as benchmarks for tracking individual player development, assessing training adequacy, and informing return-to-play protocols. Beyond individual monitoring, these composite scores provide a structured framework for workload management across positions. By using each player’s historical PC data, dynamic baselines can be established for longitudinal tracking, while position-specific thresholds allow for more tailored workload expectations. Additionally, correlations between PC components and key match events may support tactical analysis, enabling coaches to make informed decisions on training periodization, recovery strategies, and role-specific performance evaluation. This position-specific approach enhances the practical utility of PC scores in guiding performance monitoring, workload management, and injury prevention in elite rugby union environments.

## Conclusion

In conclusion, backs consistently exhibited higher training and match workload than forwards, with the front row showing the lowest values across various metrics. Inside and outside backs displayed greater demands in acceleration, deceleration, and high-speed running, while scrum-halves recorded the highest match workload in metrics such as m·min^-1^ and PL·min^-1^. Forwards, particularly the back row, had higher physical involvement in terms of tackles and carries, while backs excelled in meters carried and high-speed efforts. PCA revealed two key components of training workload: one for high-intensity explosive workload and another for low-intensity continuous workload. Similarly, PCA identified two critical components for match performance: one representing overall performance metrics, and another focusing on time-based workload measures such as TL and PL.
